# Case Report: Spontaneous pregnancy after fertility-preserving treatment in a patient with low-grade endometrial stromal sarcoma and literature review

**DOI:** 10.3389/fonc.2025.1572914

**Published:** 2025-10-08

**Authors:** Manrong Wang, Pengfei Wu, Lulu Wang, Sijia Liu, Qujia Gama, Qiaoying Lv, Jinyu Zhang, Min Yu, Yiqin Wang, Fenghua Ma, Weiwei Shan, Xuezhen Luo

**Affiliations:** ^1^ Department of Gynecology, Obstetrics and Gynecology Hospital of Fudan University, Shanghai, China; ^2^ Shanghai Key Laboratory of Female Reproductive Endocrine-Related Diseases, Shanghai, China; ^3^ Department of Assisted Reproduction, Obstetrics and Gynecology Hospital of Fudan University, Shanghai, China; ^4^ Department of Pathology, Obstetrics and Gynecology Hospital of Fudan University, Shanghai, China; ^5^ Department of Imaging, Obstetrics and Gynecology Hospital of Fudan University, Shanghai, China

**Keywords:** low-grade endometrial stromal sarcoma (LGESS), fertility-preserving treatment, hormone therapy, spontaneous pregnancy, recurrence

## Abstract

**Introduction:**

Low-grade endometrial stromal sarcoma (LGESS) is a rare malignant tumor of the uterus, characterized by slow growth. Early-stage LGESS is associated with favorable survival, but it has a high recurrence rate. The primary treatment for this disease is full-staging surgery. In this report, we present a case to explore the potential for fertility-preserving treatment in young women with LGESS.

**Case presentation:**

A 29-year-old nulliparous patient diagnosed with stage IA LGESS underwent conservative treatment at the Obstetrics and Gynecology Hospital of Fudan University. After fertility-sparing surgery and three months of hormone treatment, no residual lesions were found. Subsequently, the patient conceived spontaneously and successfully delivered a healthy baby. However, she experienced recurrence eight months after delivery but declined hysterectomy and follow-up care.

**Conclusions:**

Currently, there is still no standard management protocol for fertility preservation therapy in LGESS. Both previously reported cases and our case suggest that fertility-sparing treatment may be an option for carefully selected patients with LGESS. Further research and larger clinical studies are necessary to explore fertility-preserving treatments for young nulliparous patients with LGESS to establish guidelines or consensus.

## Background

Low-grade endometrial stromal sarcoma (LGESS) is a rare uterine malignancy, accounting for approximately 1% of all uterine malignancies (1). LGESS typically occurs in perimenopausal women but can also occur in young women and adolescents (2). It is characterized by cells similar to proliferative-phase endometrium cells, which exhibit infiltrative growth into the myometrium and/or lymphovascular spaces (3). The standard treatment for LGESS is total hysterectomy and bilateral adnexectomy (4); however, this radical surgery results in complete loss of fertility for young nulliparous females.

Around two-thirds of patients with LGESS are diagnosed at stage I-II, with a 5-year overall survival rate of 80-100% (5). Patients with stage I LGESS confined to the uterus typically have a favorable prognosis. The 5-year and 10-year overall survival rates exceed 90%, with a recurrence rate of about 10-20%. Recurrence may occur 10 to 30 years after initial treatment, reflecting the low proliferative characteristics of tumors. Considering the favorable prognosis and low proliferative characteristics of stage I LGESS, conservative treatment has been attempted in patients desiring fertility preservation, and limited cases have been reported (6–31).

Here we present a case of LGESS in which the patient conceived spontaneously and successfully delivered a healthy baby after conservative treatment. Although the disease recurred during subsequent follow-up, it still provided valuable therapeutic experience for young LGESS patients with reproductive needs and reinforced the importance of managing conservative treatment.

## Case presentation

**Figure 1:**
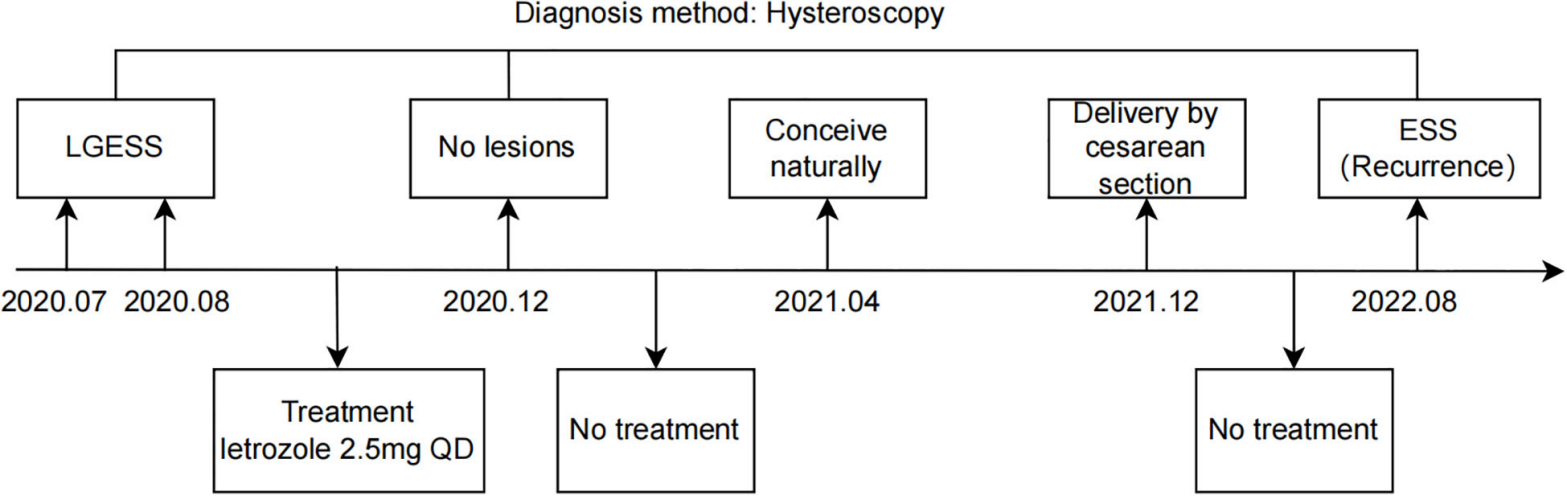
Timeline overview. LGESS, low-grade endometrial stromal sarcoma; ESS, endometrial stromal sarcoma.

The 29-year-old nulliparous female presented with LGESS and conceived naturally, ultimately giving birth to a neonate after undergoing fertility-sparing treatment. The treatment timeline is shown in **Figure 1**. She initially sought care at a local hospital due to prolonged menstruation and menorrhagia. Pelvic examination revealed a mass measuring approximately 4cm in diameter, filling the upper 2/3 of the vagina, with a palpable tip originating from the cervix and yellow discharge on the surface. Transvaginal ultrasound only suggested endometrial heterogeneity and a left ovarian cyst with no other specific signs. Pelvic magnetic resonance imaging (MRI) indicated an abnormal signal measuring about 37mm*28mm in the cervix and upper vagina, connected to the uterine cavity by its tip, initially suspected to be a submucosal leiomyoma. Subsequently, the patient underwent cervicovaginal mass excision and hysteroscopic myomectomy at a local hospital. The vaginal mass was first clamped and extracted, followed by hysteroscopic excision of the tumor-like tip remnant on the left wall of the uterine cavity. Then, the patient was referred to the Obstetrics and Gynecology Hospital of Fudan University when paraffin pathology suggested that the intrauterine mass was an endometrial stromal tumor with gonad-like differentiation in August 2020. Pathological consultation confirmed that the mass in the uterine cavity was LGESS with smooth muscle differentiation, and no relationship between the tumor and the surrounding normal muscle layer was seen. Immunohistochemistry demonstrated positive expression of estrogen receptor (ER), progesterone receptor (PR), D10, cyclin D1, and negative expression of caldesmon in the lesion. Additionally, the Fluorescence *In Situ* Hybridization (FISH) technique detected no abnormalities in the JAZF1 gene.

Despite recommendations for hysterectomy following diagnosis confirmation, she opted for fertility-sparing treatment after thorough consideration of associated risks along with her family members. Risks include the fact that fertility-preserving treatment is not the standard treatment and carries the danger of treatment failure, disease progression, and even life-threatening conditions, as well as a high risk of recurrence and pregnancy failure, even if the disease is in complete remission after treatment. All parties were fully informed about these and provided informed consent accordingly. Before starting fertility-sparing therapy, a comprehensive assessment was conducted, encompassing the patient’s medical history, family history, metabolic condition, pelvic examination, ultrasound scanning, enhanced pelvic MRI, enhanced abdominal CT, and hysteroscopic evaluation. The patient underwent laparoscopic myomectomy combined with hysteroscopic removal of endometrial polyps three years ago and has no family history of tumors. The patient’s body mass index (BMI) is 25.2kg/m^2^, and blood pressure (BP) is within normal range. Fasting blood examination revealed no abnormalities in liver and renal functions, fasting lipids, serum tumor biomarkers, or blood glucose; however, there was an increase in fasting insulin (FINS). Homeostasis model assessment-insulin resistance (HOMA-IR) was calculated as FBG (mmol/L) * FINS (μU/mL)/22.5, which indicated insulin resistance with a HOMA-IR value of 5.84 but no signs of metabolic syndrome (MS). Hormonal levels, including AMH, estradiol, progesterone, testosterone, follicle-stimulating hormone (FSH), and luteinizing hormone (LH), were within normal limits. Breast ultrasound suggested bilateral breast nodules with BI-RADS grade 3. Abdominal enhanced computed tomography (CT) indicated a small stone in the left kidney requiring follow-up. Pelvic enhanced MRI showed heterogeneous signal in the myometrium, which may be residual lesions or adenomyosis, along with an endometriotic cyst of the left ovary. No extrauterine lesions or metastasis were found.

Currently, there is no consensus on the optimal therapy regimen for conservatively treating LGESS in terms of drug choice, dosage, or duration. Following a multidisciplinary discussion in September 2020, the patient was suggested to take letrozole at a dose of 2.5 mg orally once daily for 6 months. Referring to the follow-up strategy for patients with early endometrial cancer, we suggested that these patients should undergo hysteroscopy every 3 months during treatment, and that the frequency of the review could be relaxed to every 6 months after 2 consecutive postoperative surgeries in which the pathology did not show any lesions. After three months of treatment, the patient underwent a hysteroscopy to assess endometrial pathology, and no residual tumor lesions were found. A pelvic MRI examination was also performed, which did not indicate any lesions. Subsequently, the patient discontinued her medication by herself. Four months later, the patient became pregnant spontaneously after letrozole ovulation induction and underwent a cesarean section in December 2021 at a local hospital due to preterm labor and a history of multiple hysteroscopic procedures. She ceased breastfeeding one month after delivery and menstruated seven months later.

In August 2022, the patient reported experiencing reduced menstrual flow after a menstrual transition and visited our hospital. Pelvic MRI indicated an irregular signal in the left uterine horn. The patient immediately underwent a hysteroscopic assessment, indicating an endometrial stromal tumor, implying disease recurrence. Surgery was recommended immediately; however, the patient refused and was subsequently lost to follow-up.

## Literature review

In order to further investigate the feasibility of fertility-sparing management in LGESS, we conducted a review of 26 available English literature sources. These sources included 102 cases of conservative treatment (**Table 1**), with the majority being at stage I, as well as 2 cases at stage II and 3 cases at stage III (excluding those not described in detail in the study). Many patients are accidentally diagnosed with LGESS after hysteroscopic or laparoscopic lesion removal, and then opt for conservation therapy because of their young age or strong fertility needs. The youngest patient was only 14 years old, as reported by Zheng et al. in 2020 (13). According to the data, out of the 102 patients who received fertility-sparing treatment, the median age was 29 years (range:14–40), and the follow-up time ranged from 3 to 240 months. After the fertility-sparing surgery, including hysteroscopic, laparoscopic, or transabdominal lesion resection, 69.9% (58/83) of them received hormone therapy. Among the 102 patients, 46.1% (47/102) were able to conceive, with a total of 33.3% (34/102) patients successfully delivering. The recurrence rate was 60.8% (62/102), with two patients surviving with the disease (Final follow-ups were 24 and 240 months, respectively), and two patients died ten years after the conservation treatment.

**Table 1:** Literature review of LGESS fertility-sparing management.

Year	Reference	Case no.	Age	Stage	Immunohist ochemistry	Treatment	Treatment duration (months)	Recurrence (months)	Hysterectomy	Pregnancy	Status, follow-up (months)
2024	Rajaram et al. (6)	1	22	IA	ER:+; PR:++; BCOR-; Ki:10~12%	GnRHa 3.75mg/4w	6	No	/	/	NED(6)
		2	19	IB	ER+; PR+; CD10+; desmin+; BCOR+; cyclin D1	GnRHa 3.75mg/4w	6	No	/	/	NED(6)
2023	Laufer et al. (7)	1	33	IA	ER+; PR+; CD10+; desmin+	progesterone+LNG-IUD	3 and 4	Yes(7)	TH/BSO	NPTD	NED(36)
2023	Yano et al. (8)	1	34	IIB	ER+; CD10+; α-SMA+; desmin-	No	/	Yes(24)	TH/BSO	/	NED(16)
2022	Piątek et al. (9)	1	35	/	/	MA 160 mg/d	12	No	/	NFTD	NED(24)
		2	29	/	/	MA 160 mg/d	12	Yes(46)	/	/	NED(46)
		3	34	/	/	MA 160 mg/d	12	Yes(35)	/	/	NED(90)
2022	Huang et al. (10)	23	29 (15-40)	IA 7/23 IB 12/23 I 3/23 IIIB 1/23	ER/PR +22/23 No rep 1/23	High-dose Progestins 7/23 Non-progestin 9/23 No 7/23	≤6 months 11/16 > 6months 5/16	Yes(15/23)	TH 7/15	Delivery 6/23 Ongoing pregnancy 1/23 Abortion 1/23	NED 24 (3-107)
2021	Gu et al. (11)	1	28	IB	ER+;PR+; CD10+; desmin+	No	/	Yes(70)	/	NFTD	NED(96)
2020	Xie et al. (14)	1	32	IA	CD10+Ki-67 (1%, +); Inhibin-α-; Calretinin-; Desmin-	CM	15	No	/	NFTD	NED(35)
2020	Zheng et al. (13)	1	27	IB	ER+;PR+; CD10+; desmin-	MPA 500 mg/d and GnRHa 3.75 mg/4 w	12 and 6	Yes(22)	TH/BSO	/	NED (22)
		2	15	IIB	ER++;PR++; CD10+; desmin-	MPA 500 mg/d	12	Yes(31)	/	/	NED(31)
		3	14	IB	ER+; PR+; CD10+;	MA 160 mg/d	12	No	/	/	NED(74)
		4	19	IIIB	ER++; PR+++; CD10+;	MPA 250 mg/d	12	Yes(56)	TH/BSO	NFTD	NED(56)
		5	24	IB	ER+++; PR+++; CD10+; desmin+	MPA 250 mg/d	12	Yes(45)	TH/BSO+CRS	NFTD	NED(45)
2018	Chin et al. (15)	1	34	IB	ER 50%; PR 80%; CD10 +++; CD31-; CD34-; SMA-	MA 160 mg/day	48	Yes(84)	TH/BSO	/	NED
2017	Xie et al. (12)	17	28(15-37)	IA 6/17 IB 11/17	ER+ 15/17 PR+ 17/17	MA/MPA 9/17 GnRHa 4/17 GnRHa+L NG-IUD 2/17 No 2/17	/	Yes(IB,10/11 4-106 M)	FSS 4/10 TH/BSO 2/10 TH 1/10 CRS 3/10	NFTD 4/5 NPTD 1/5	NED 39(4-106)
2015	Maeda et al. (16)	1	24	/	ER++; PR++; CD10+	No	/	Yes(10)	CRS	NFTD	AWD (> 240)
2015	Noventa et al. (17)	1	34	IB	ER+++; PR+++; CD10+; SMA+; vimentin+; desmin-; h- caldesmon-	No	/	No	/	Pregnant at 11 weeks	NED(19)
2015	Morimoto et al. (18)	1	25	/	ER+; PR+; Ki-67<5%	MPA 600 mg/d	39	Yes(12)	TH/BSO	/	DOD(> 124)
2015	Jin et al. (19)	1	36	IA	/	MA 320 mg/d	5	No	/	NFTD	NED(38)
		2	28	IB	/	MA 160 mg/d	6	No	/	NFTD	NED(40)
		3	37	IA	ER: 80%++; PR: 90%+++	MA 160–320 mg/d	6	No	/	NFTD	NED(24)
		4	32	IB	ER+++; PR+++	GnRHa 3.75 mg/4w	5	No	/	/	NED(39)
		5	29	IB	ER ±; PR +	MA 320 mg/d	3	Yes	TH	/	NED(15)
2015	Laurelli et al. (20)	1	38	IA	ER+; PR+; CD10+; desmin+	No	/	No	/	NFTD	NED (70)
		2	33	IA	ER+; PR+; CD10+; desmin+	MA 160 mg/d	12	No	/	SFTM	NED (54)
		3	40	IA	ER+; PR+; CD10+; desmin+	MA 160 mg/d	24	No	/	NFTD	NED (48)
		4	18	IA	ER+; PR+; CD10+; desmin+	MA 160 mg/d	24	No	/	/	NED (39)
		5	34	IA	ER+; PR+; CD10+; desmin+	MA 160 mg/d	24	No	/	/	NED (32)
		6	30	IA	ER+; PR+; CD10+; desmin+	MA 160 mg/d	24	No	/	/	NED (30)
2014	Jain et al. (21)	1	23	IB	/	No	/	Yes(12)	CRS	NFTD	NED (54)
2014	Bai et al. (22)	19	/	/	/	/	/	Yes(15/19,20.5M)	/	SFTM 8/19 NFTD 5/19	/
2014	Choi et al. (23)	1	31	IA	ER++; PR++; CD10++	Letrozole 2.5mg/d	6	No	/	NPTD	NED (99)
2014	Zhan et al. (24)	1	26	IB	SMA+; CD10-	CT+MPA	7	No	/	NFTD	NED(47)
2014	Dong et al. (25)	1	19	III	ER+++; PR+++; CD10+; desmin+; SMA-; Ki-67 30%; p53-	MPA	12	No	/	/	NED(33)
2012	Delaney et al. (26)	1	16	IB	/	MA	96	No	/	NPTD	NED (108)
2012	Sánchez-Ferrer et al. (27)	1	32	IB	ER+; PR+;CD10+; SMA+; Ki-67 <10%;	MA	16	Yes(30)	TH	NPTD	NED(60)
2009	Koskas et al. (28)	1	34	IA	ER+++; PR+++; Ki-67 5%; p53-; CD10+++	No	/	Yes(10)	/	NFTD	AWD(24)
2005	Stadsvold et al. (29)	1	16	IB	ER++; PR++	MA	21	No	/	/	NED(21)
1997	Lissoni et al. (30)	5	27(18-36)	/	/	No	/	No	/	SFTM(1/5)	NED 51(12-84)
1990	Mansi et al. (31)	1	24	I	/	No	/	Yes(66)	/	/	DOD (120)
	Total	102 cases	29 (14-40)	I(72/77 93.5%) II(2/77, 2.6%) III(3/77, 3.9%)		Progesterone (39/83,47.0%) GnRHa (19/83,22.9%) Observation (22/83,26.5%)	12(3-96)	Recurrence rate:60.8% (62/102)		Pregnancy rate:46.1% (47/102)	NED (3-107) AWD (24 to more than 240) DOD (more than 124)

LGESS, low-grade endometrial stromal sarcoma; ER, estrogen receptors; PR, progesterone receptors; SMA, smooth muscle actin; BCOR, BCL6 corepressor; MPA, medroxyprogesterone acetate; GnRH-a, gonadotrophin-releasing hormone analogues; MA, megestrol acetate; CM, Chinese Medicine; LNG-IUD, levonorgestrel-releasing intrauterine device; TH/BSO, total hysterectomy and bilateral salpingo- oophorectomy; TH, total hysterectomy; CRS, cytoreductive surgery; FSS, fertility-sparing surgery; SFTM, spontaneous first-trimester miscarriage; NFTD, normal full-term delivery; NPTD, normal preterm delivery; NED, no evidence of disease; DOD, dead of disease; AWD, Alive with disease.

Summarized data show treatment options including high-dose progestins, aromatase inhibitors, and gonadotropin-releasing hormone analogues (GnRH-a). The most commonly used progestin is megestrol acetate (MA) at a dose of 160–320 mg per day for six months to two years (13, 19, 20). Additionally, Rajaram et al. (6) and Choi et al. (23) reported treatment regimens of GnRH analogue 3.75 mg every 4 weeks intramuscularly for 6 months and letrozole 2.5 mg daily orally for 6 months, respectively, with no recurrence in any of the patients.

According to previous case reports, patients who chose conservative treatment had a high recurrence rate, ranging from 58.8% to 83.3%, while some other reports indicated a lower range of 0-20% (19, 20). Huang et al.’s study included a total of 153 patients (10), with 23 in the conservation therapy group, which had the largest sample size as shown in **Table 1**. In the fertility-sparing cohort, 69.6% (16/23) of patients received postoperative hormone therapy, including high-dose progestins and GnRH-a, with a recurrence rate of 65.2% (15/23) and a median disease-free survival (DFS) of 24 months. Despite nearly all patients being at stage I and expressing positive ER/PR, the prognosis was significantly worse compared to the cohort without fertility-sparing, where about 70% of patients were at stage I but had a lower recurrence rate of 17.7% and a median DFS of 47 months. 34.8% (8/23) of these patients conceived, with 26.1% (6/23) delivering successfully, 4.3% (1/23) experiencing a miscarriage, and 4.3% (1/23) being in the process of pregnancy. However, no statistically significant difference was found between radical surgery and local tumor excision in terms of overall survival (13), suggesting that although recurrence rates increase, fertility-sparing management may not affect overall survival.

Among patients undergoing conservative treatment, the pregnancy rate ranged from 29.4% to 60%. Koskas et al. (28) reported the first successful pregnancy in a patient undergoing conservation therapy, who conceived rapidly but had severe peritoneal recurrence in the postpartum period. In the study by Huang et al., nearly half of the patients chose hysterectomy after the first recurrence, with this proportion eventually reaching 80% after repeated recurrence, and only two of those patients had given birth before undergoing hysterectomy. Instances of disease progression and fatal cases were also reported, highlighting the dual risk of failed pregnancy and disease advancement during conservative treatment.

## Discussion

Here we present a case of LGESS in which the patient conceived naturally and successfully delivered after conservative treatment, but experienced a relapse 8 months postpartum. According to the guidelines (4), tumor staging remains the most important prognostic factor for LGESS. Given the favorable oncologic outcomes associated with early-stage LGESS and successful cases of conservative therapy, fertility-sparing treatment may be considered for patients who strongly desire to preserve fertility. However, the high recurrence rate remains a critical problem after conservation therapy, and decisions regarding conservation therapy must be made carefully following a multidisciplinary consultation. The above literature review and our report also confirm the feasibility of reproductive organ preservation for patients with LGESS and emphasize the importance of recurrence prevention after completing childbearing.

Careful evaluation and accurate diagnosis are crucial before initiating treatment. The ultrasound presentation of LGESS is nonspecific, typically characterized by heterogeneous hypoechoic endometrial masses that may involve extensive myometrium. On MRI imaging, these tumors usually appear as large masses with or without myometrial invasion (32, 33), lacking disease-specific features, so they are often misdiagnosed as leiomyoma or adenomyosis. It is challenging to distinguish LGESS from other malignancies based solely on imaging. A comprehensive hysteroscopic examination is necessary to assess lesions in the uterine cavity. Our case was not exceptional in terms of imaging results; therefore, histopathology remains essential for diagnosing LGESS.

LGESS is a low-grade sarcoma with metastatic potential. It consists of homogeneous cells similar to proliferative endometrial stromal cells, often forming distinctive finger-like projections that invade the myometrium, veins, and lymphatics. Its histological features include dense, homogeneous stromal cells with inconspicuous cellular pleomorphism, mild nuclear atypia, and variable mitotic figures. Additionally, it exhibits various morphological features such as fibrous or mucoid changes, and glandular, smooth muscle, or sex cord-like differentiation (34). The pathology of our case suggests an endometrial stromal tumor with smooth muscle differentiation. For tumors with focal smooth muscle differentiation, if the smooth muscle component is less than 30% of the total volume, the tumor is classified as LGESS; if the smooth muscle component is higher, the tumor is referred to as a mixed endometrial stromal and smooth muscle tumor (1). Due to the morphological diversity of LGESS, precise pathological diagnosis is vital, especially for young nulliparous cases seeking fertility preservation. Besides, positive expression of estrogen receptor (ER), progesterone receptor (PR), and CD10 was preserved in the majority of LGESS cases. Patients with ER/PR positive expression typically respond well to hormonal therapy, and this over-expression of hormonal receptors appears to have significant prognostic value for overall survival (OS) (35). Therefore, immunohistochemistry is currently utilized as an aid in diagnosis and selection of hormonal drugs. In our case, the patient exhibited ER/PR positive expression, suggesting the feasibility of hormone therapy.

Additionally, cytogenetic analyses revealed the presence of multiple repeated non-random chromosomal translocations in patients with LGESS. The most common translocation involves the fusion of two zinc finger genes, JAZF1 and JJAZ1, resulting from a translocation between the short arm of chromosome 7 and the long arm of chromosome 17. This characteristic fusion gene was found in more than 50% of LGESS cases but has not been observed in other uterine sarcomas or smooth muscle tumors (36–38). Other associated genetic abnormalities include JAZF1/PHF1, EPC1/PHF1, and MEAF6/PHF1 (39, 40). Our study utilized the FISH technique to test for the presence of JAZF1/JJAZ1 gene abnormalities; however, none were detected. Due to technical limitations, we did not examine the rest of the genes. Jan Hojný et al. constructed the largest molecular map of uterine sarcomas to date by RNA sequencing clustering analysis of 262 cases of uterine sarcomas, including LG-ESS, HG-ESS, UUS, and UTROSCT, combined with immunohistochemistry (IHC) and DNA mutation detection. Further analysis showed that LG-ESS was divided into two expression subgroups regardless of the presence of fusion genes such as JAZF1::SUZ12, and the results show that fusion genes were associated with different overall and recurrence-free survival outcomes, with the fusion-negative group having a better prognosis (41). The involvement of the proteins encoded by these fusion genes in tumorigenesis and progression, as well as the significance of the results on the fusion genes for guiding clinical therapy, has not been previously reported. We anticipate further basic research to guide clinical treatment and prognosis prediction.

After a precise diagnosis and comprehensive assessment, it is crucial to select appropriate treatment options for patients without contraindications to conservation. Recommendations for the conservative treatment of patients are now mainly based on previously reported cases as well as conservation treatment protocols for early-stage endometrial cancer (42).

LGESS is a rare tumor that is hormone-dependent. According to current international guidelines (NCCN, ESMO), hormone treatment is considered an essential part of managing LGESS (4, 43). However, there have been no prospective studies published regarding the role of hormone therapy. Nevertheless, adjuvant hormonal therapies have been shown to decrease the recurrence rate in clinical trials (44). Limited and retrospective data suggest MA is the most commonly used drug, with GnRHa and letrozole also cited.

Despite the favorable prognosis for early-stage LGESS, it is essential to acknowledge that a positive outcome for patients undergoing conservative therapy cannot be assumed, and individualized decisions are still necessary. While overall survival does not seem to be impacted in these cases, it is important to emphasize that individuals opting for fertility-sparing surgeries should be encouraged to conceive as soon as possible and consider radical surgery after completing childbearing due to the higher rates of recurrence and potential disease progression. For those who are determined to retain their uterus, intensive follow-up should be conducted, and related risks should be fully informed.

In our present case study, we used Letrozole as an aromatase inhibitor following fertility-sparing surgery (45, 46); the patient conceived spontaneously, delivered successfully, but experienced a recurrence detected at 8 months postpartum. Upon retrospective review of this case, it is speculated that several factors may have contributed to the positive outcome. Firstly, the patient presented with Stage IA (12) LGESS characterized by a localized lesion and no myometrium invasion. Secondly, aside from insulin resistance, the patient did not have any complications or other infertility factors. Thirdly, adjuvant hormone treatment was administered based on existing literature due to the lack of available standards for reference. Furthermore, there were no identified infertility factors present in the patient’s partner. The patient’s successful conception and delivery after treatment were gratifying, but a recurrence occurred eight months after delivery. We summarize three possible reasons for the patient’s relapse in a short period. Firstly, the biological characteristics of sarcomas. According to the available data, the recurrence rate of LGESS treated with nursery was as high as 60.8% (62/102), which may suggest that although LGESS is an indolent-growing uterine sarcoma, its aggressiveness as a uterine sarcoma should not be ignored, especially in nursery patients who did not undergo radical surgery. Secondly, patient compliance is the most important thing in nursery treatment. For patients opting for conservation treatment, radical hysterectomy is preferred after completion of childbearing, and those who refuse surgery should continue medication and be strictly followed up. In this case, the patient was not on medication and was not reviewed between the time of successful delivery and the onset of recurrent symptoms. This indicated that patient compliance with strict, regular surveillance and treatment is the foundation of conservation therapy. Thirdly, the influence of pregnancy-related hormones. There is no clear evidence that pregnancy and hormonal changes directly lead to the recurrence or progression of LGESS. Koskas et al. reported a case in which a patient with LGESS was naturally conceived after receiving nursery treatment, but severe peritoneal recurrence occurred after delivery. This all suggests that dramatic hormonal fluctuations in the body during pregnancy and postpartum, mainly a significant increase in estrogen and progesterone levels and a sharp decrease after delivery, may promote the development of LGESS, but the exact mechanism is not clear.

In summary, early-stage LGESS is an indolent malignancy with generally favorable survival outcomes. Fertility-sparing management may be considered by those who desire to preserve their reproductive potential, since the overall survival does not seem to be impacted in the group of patients who opted for fertility preservation therapy in the previous cases, despite the high recurrence rate. Consequently, after thorough evaluation, the decision for fertility-preserving treatment may be feasible; this decision should be made by multidisciplinary specialists such as gynecological oncologists and gynecological pathologists. Patients should be fully informed about the likelihood of fertility failure and the increased risk of recurrence after conservative therapy. Therefore, how to improve pregnancy and live birth rates in patients treated with conservation therapy, reduce recurrence rates, and respond to disease progression during treatment in a timely and effective manner are all urgent issues to be addressed. Multicentric prospective studies with larger sample sizes are needed to provide high-quality evidence on conservative management strategies.

## Data Availability

The original contributions presented in the study are included in the article/supplementary material. Further inquiries can be directed to the corresponding authors.
